# Increasing human environmental footprint does not lead to biotic homogenization of forest bird communities in northern USA

**DOI:** 10.1002/ece3.10015

**Published:** 2023-04-19

**Authors:** Eric Le Tortorec, Matti Häkkilä, Edmund Zlonis, Gerald Niemi, Mikko Mönkkönen

**Affiliations:** ^1^ Department of Biological and Environmental Sciences, Faculty of Mathematics and Science University of Jyvaskyla Jyvaskyla Finland; ^2^ School of Resource Wisdom University of Jyvaskyla Jyvaskyla Finland; ^3^ Natural Resources Research Institute Duluth Minnesota USA

**Keywords:** alpha‐diversity, beta‐diversity, gamma‐diversity, habitat loss, human footprint index

## Abstract

Studies have shown negative impacts of increased human pressures on biodiversity at local (alpha‐diversity) and regional (gamma‐diversity) scales. However, the diversity between local sites (beta‐diversity) has received less attention. This is an important shortcoming since beta‐diversity acts as a linkage between the local and regional scales. Decreased beta‐diversity means that local sites lose their distinctiveness, becoming more similar to each other. This process is known as biotic homogenization. However, the mechanisms causing biotic homogenization have not been fully studied nor its impacts on different facets of biodiversity. We examined if land‐use change due to human actions causes biotic homogenization of taxonomic, functional, and phylogenetic diversity in bird communities of forested habitats in the state of Minnesota, USA. We address if forest loss and increased human domination in a region were associated with decreased beta‐diversity. Our results showed that elevated human pressure was not related to increased biotic homogenization in this study region. Effects of landscape change were incongruent among taxonomic, functional, and phylogenetic diversity. At all spatial scales, taxonomic diversity was unrelated to forest loss or human domination. Interestingly, increased human domination appeared to increase the functional beta‐diversity of bird communities. This association was driven by a decrease in local diversity. Forest habitat loss was associated with decreasing functional and phylogenetic diversity in local communities (alpha‐diversity) and in regional species pool (gamma‐diversity), but not in beta‐diversity. We highlight the importance of considering multiple facets of biodiversity as their responses to human land‐use is varied. Conservation significance of beta‐diversity hinges on local and regional diversity responses to human land‐use intensification, and organization of biodiversity should therefore be analyzed at multiple spatial scales.

## Graphical abstract

We address if increased human land‐use intensity results in biotic homogenization, that is, decreased variation in structure among local sites, in forest bird communities. In this analysis, we used bird census data from Minnesota, USA. Elevated human pressure was not related to increased biotic homogenization but interestingly, increased human pressure appeared to increase the between‐site functional diversity of bird communities.

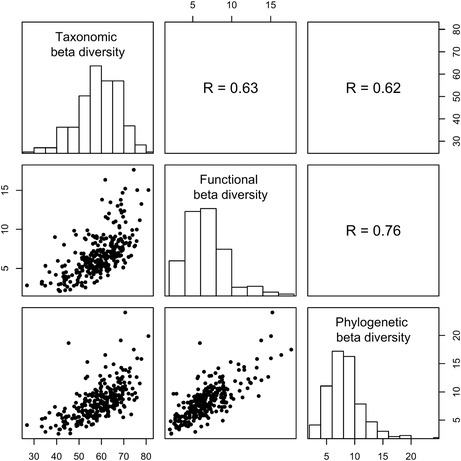

## 1 INTRODUCTION

Global studies have shown that land‐use changes and their associated pressures have led to strong, consistent, and accumulating negative effects on biodiversity (Díaz et al., 2019; Haddad et al., 2015). Human pressures that contribute to land‐use change such as agriculture, timber harvesting, and urbanization have significantly modified ecosystems worldwide. Of these, forest ecosystems have been especially heavily impacted by human activities, facing alarming rates of both deforestation and forest degradation (Curtis et al., 2018; FAO & UNEP, 2020). Forests play an essential role in mitigating and adapting to climate change. The fight against climate change is considered requiring increasing amounts of forest‐based energy and bioproducts for the purposes for which we use oil, coal, and gas today (Hetemäki et al., 2022). Thus, pressures on remaining forests are continuously increasing and forest degradation due to forestry will likely further accelerate in future. Natural forests harbor high levels of productivity, biomass, and biodiversity and decreases in their cover potentially could have significant negative impacts on biodiversity and ecosystem services (Chase et al., 2020; Foley et al., 2005; Matricardi et al., 2020). Betts et al. (2022) suggested that forest degradation drives widespread avian habitat and population declines in boreal forests and may therefore be a primary cause of biodiversity decline in managed forest landscapes. However, our understanding is more limited on how forest habitat loss and degradation affects different aspects of biodiversity (taxonomic, functional, and phylogenetic) and at what spatial scale.

Human activities appear to impact different scales of biodiversity in different ways (McGill et al., 2015). Studies have shown that biodiversity at the local scale (alpha‐diversity) has not changed significantly through time, whereas biodiversity at the global scale has decreased. For example, Vellend et al. (2013) showed that local‐scale plant species diversity has not significantly changed through time and that species increases were as common as species decreases. Similarly, Dornelas et al. (2014) showed that time series of local species richness did not show a systematic loss, although community composition changed. These two meta‐analyses collectively analyzed >250 individual data sets on biodiversity change through time and showed that local assemblages are experiencing a substitution of their taxa, rather than systematic loss. This raises a question whether the substitution process is associated with changes in similarity among local communities (beta‐diversity). Still globally, many taxa, key habitats and ecosystems are at risk. Newbold et al. (2015) inferred from models based on empirical data that at a global scale, local assemblages have lost over 13% of their species richness due to human pressures. The discrepancy between trends in local and global biodiversity can be explained by a spatial measure of biodiversity—beta‐diversity.

Beta‐diversity measures variation in biodiversity in space and is derived from the total diversity of a region (gamma‐diversity) and the local diversities of sampled sites (alpha‐diversity). Beta‐diversity, in its additive form, is calculated as gamma‐diversity minus the mean alpha‐diversity of a region (Tuomisto, 2010). In this form, beta‐diversity reflects absolute effective species turnover by quantifying how much the species richness of an entire region exceeds that of an average single location. Thus, even though local diversity might not show a decrease, the total diversity of a region could decrease if the individual sites became more similar in composition. For example, uneven losses in species between sites can lead to decreased mean alpha‐diversity, which will increase the beta‐diversity of a region. On the other hand, the colonization of specialist species into new sites can increase mean alpha‐diversity (McCune & Vellend, 2013) and result in a decrease in the beta‐diversity of a region. This holds for both the additive and multiplicative forms of beta‐diversity. Thus, in a conservation setting, maximizing beta‐diversity is not necessarily desirable for the conservation of gamma‐diversity (Socolar et al., 2016).

Increasing similarity between local sites (decreased beta‐diversity) is a process known as biotic homogenization (Olden, 2006). Biotic homogenization has been shown to be a global phenomenon, occurring in most taxonomic groups (Baiser et al., 2012) and commonly results from human land‐use intensification such as urbanization (McKinney, 2006), intensive agriculture (Ekroos et al., 2010;), and forest loss (Ibarra & Martin, 2015). Gossner et al. (2016) concluded that biotic homogenization rather than local diversity loss could be the most important consequence of human land‐use intensification. Human activities are associated with the introduction of exotic species, which replace native species and result in different sites to become more similar (Baiser et al., 2012; Finderup Nielsen et al., 2019). Local species extinctions often include specialists with narrow dietary and habitat preferences and are replaced by generalists better adapted to use a wider variety of resources and survive fluctuating environmental conditions within human‐built environments (Clavel et al., 2011; McKinney & Lockwood, 1999). These generalists often have broad regional or global ranges (La Sorte & Boecklen, 2005). Specialists, by definition, possess specific functions, and their loss in a community decreases functional diversity. Functional diversity is a concern for human well‐being because it measures essential life components in the ecosystem such as pollination or decomposition (Cardinale et al., 2012). There is already evidence that the loss of biodiversity has created an ecosystem service debt and lowered resilience that will be increasingly exacerbated by continued biodiversity loss (Isbell et al., 2015; Oliver et al., 2015). Furthermore, specialized species often have unique phylogenetic histories, the loss of which decreases the phylogenetic diversity and, thus, the capacity of the biological community to evolve to future environmental change. Biotic homogenization primarily has been studied from the perspective of species richness or taxonomic diversity, but we have a limited understanding of how other facets of biodiversity (e.g., functional or phylogenetic) respond to human activities.

We studied whether land‐use change due to human actions is associated with biotic homogenization in bird communities of forested habitats in the state of Minnesota, USA. We used Minnesota Breeding Bird Atlas based on bird point‐count data. Our aim was to examine whether increasing human footprint and changes in regional forest cover (habitat loss) are associated with decreased beta‐diversity as predicted by biotic homogenization theory. Human footprint includes human population density, land‐use change, transportation infrastructure, and electrical power infrastructure (Sanderson et al., 2002). Different aspects of human footprint index are strongly correlated with each other (Sanderson et al., 2002; Appendix S1), and their independent contributions to biotic homogenization is therefore difficult to evaluate. For example, transportation infrastructure (roads) fragment landscapes and trigger human colonization and degradation of ecosystems, and the presence of roads is highly correlated with changes in species composition, including increases in non‐native invasive species, decreased native species populations through direct and indirect mortality (Crist et al., 2005; Ibisch et al., 2016). We considered human footprint a generic measure of human domination or the level of human disturbance and predicted that increased human footprint would lead to decreased beta‐diversity. Forest loss has shown to homogenize local communities (Ibarra & Martin, 2015) but its effects on beta‐diversity depend on how selectively species are lost from the system. Because changes in beta‐diversity may result from changes in regional (gamma) diversity, in local (alpha) diversity, or both, we estimated regional and local diversities as well. While our focus is on effects of forest loss and human footprint on diversity, we controlled for factors known to impact biodiversity, including habitat diversity (positive effect on regional species richness), net primary productivity (positive effect on diversity at several spatial scales) and temperature (positive effect) (Honkanen et al., 2010; Qian, 2010). We examined alpha‐, beta‐, and gamma‐diversity from taxonomic, functional, and phylogenetic perspectives, which are expected to show varied responses to changes in human influence (Devictor et al., 2010).

## 2 METHODS

### 2.1 Study design

Bird point‐count data were collected annually from 2009 to 2014, as part of the Minnesota Breeding Bird Atlas (MNBBA; Pfannmuller et al., 2017). The goal of the MNBBA was to systematically sample breeding birds across the state by collecting data in each township of the state. Townships measure approximately 93 km^2^ (6 × 6 miles, approximately 9.65 × 9.65 km) and were developed by the Public Land Survey System (https://mnatlas.org/resources/public‐land‐survey‐quarter‐quarter‐sections/). Within each township, three point‐count locations were selected. The first point was randomly selected. The second and third points were randomly selected in the most abundant and second most abundant cover types, respectively. All points were at least 250 m apart. This process ensured a random selection of points and sampling in distinct cover types as opposed to an extensive number of edges. Land‐use and cover‐type data were derived from the 2001 National Land Cover Database (Homer et al., 2004). Counts were conducted primarily on secondary roads; large, paved roads such as state or federal highways were avoided. Townships in roadless areas such as the Boundary Waters Canoe Area Wilderness, Voyageurs National Park, and the Red Lake Peatland were sampled from trails, portages, and water by hiking, biking, boat, or canoe, but the random point was designated the closest access point to the township. The second and third points adhered to the same process as those in road‐accessible areas.

The primary objective of the MNBBA point counts was to ensure equal and consistent sampling of the bird community across the state of Minnesota. This included a random sampling approach and standardization of effort in gathering data, plus the ability to gather data within the dominant and subdominant cover types within each township. Individuals participating in the gathering of point counts were required to pass a test of 86 bird species songs or have more than 5 years of field experience in counting birds with point counts in Minnesota. Those gathering data on point counts were also tested by audiologists to ensure their hearing ability was in normal ranges and they participated in 3 days of “standardization” in point count data gathering with an experienced field ornithologist (Niemi et al., 2016).

During a 10‐min point count, all birds seen or heard were recorded (i.e., unlimited distance) and distances from the point were estimated to allow habitat‐specific data to be used (observations within 25, 50, 100 m, and >100 m of the census point). Point counts were completed from the last week of May (in southern MN) to the second week of July (northern MN) from 2009 to 2014. Most counts were completed in June. Point counts were gathered from approximately 0.5 h before to 4 h after sunrise on days with little wind (<15 km/h) and little or no precipitation. Extensions from sunrise to 6 h were allowed for counts in western MN due to the high proportion of windy days and based on the experience of ornithologists in that region. All points were located and marked with a GPS device, cover type of vegetation visually estimated, and pictures taken in the two directions perpendicular to the road.

A total of 7070 MNBBA counts were conducted between May 2009 and June 2014. Abundances of birds identified to the species level within 100 m of the MNBBA point were used since the landcover type classification was conducted at the same spatial scale (see below). For the analyses in this study, we used MNBBA points located in forested landcover classes. To ensure that the dominant cover type being sampled was forest, we first calculated the proportion of landcover types within 100 m of each MNBBA count location using ArcMAP version 10.2.2 (ESRI, 2014) and the “isectpolyrst” tool in Geospatial Modeling Environment version 0.7.3.0 (Beyer, 2012).

Landcover classes were derived from the LANDFIRE dataset (Rollins et al., 2006). One hundred fifteen cover types in the LANDFIRE Existing Vegetation Type database were consolidated and reclassified into 25 classes representing the land‐use and cover types available in Minnesota (30 m spatial resolution). Each count location was characterized by the dominant (highest proportion) landcover type. All counts with any of the following dominant landcover classes were considered forested: boreal coniferous, boreal deciduous, lowland coniferous, lowland deciduous forest, northern hardwoods, oak forest, oak savannah, parkland deciduous forest, pine forest, pine‐oak barrens, and rural and urban developed forest (Appendix S2). For those MNBBA points counted more than once, we selected the earliest temporal observation. Occasionally, due to field sampling variation, points were located within 250 m of each other. In this scenario, we retained MNBBA points located at least 200 m from each other (twice the distance of the bird count radius) to avoid overlap between bird counts. From pairs of MNBBA points located under 200 m from each other we selected the earlier observation, and if both points had the same date, one was chosen randomly.

The townships in Minnesota were combined into 617 units, each comprising four townships and measuring roughly 19.3 × 19.3 km (12 × 12 miles). This was done to ensure a large enough number of sampling units with an adequate number of local communities. Each unit formed a square, except for a few areas where irregular units constructed from one to four townships were used. Units were developed by selecting a random township, which was used as the north‐eastern square of the first unit. Subsequent units were then formed using the first unit as a reference point until the entire state was covered. For calculating bird community diversities, we only used units that had at least 3 MNBBA points located in a forested land‐use class. We determined 3 points to be the minimum number of communities to reliably estimate beta‐diversity. This resulted in 287 sampling units (regions) with a total of 2217 forested MNBBA points (local communities), and on average, 7.2 points (std. 2.96; maximum is 18) per unit. Total number of species in the final data was 162.

Our analysis was restricted to the contemporary, primary forested areas of Minnesota. Except for the extinct Passenger Pigeon (*Ectopistes migratorius*) and the possibly extirpated Rusty Blackbird (*Euphagus carolinus*, last known nesting in 1986), there are no known extinctions or extirpations of bird species that have occurred in Minnesota's northern‐forested regions. However, there have been many examples of species breeding range contractions northward due to forest losses in the southern and western regions of the state that were not included in this analysis (Pfannmuller et al., 2023). The loss and extensive fragmentation of these forests began to occur in the 1800s and primarily affected forests in the southeastern, western, and northwestern regions of Minnesota (LCCMR, 2007).

### 2.2 Calculation of beta‐diversities

Within each unit, we calculated taxonomic, functional, and phylogenetic alpha‐, beta‐, and gamma‐diversities with Rao's quadratic entropy using R functions developed by De Bello et al. (2010). The Rao index measures dissimilarity by summing total dissimilarity and weighting it by species proportions. In addition, the Rao index makes it easy to incorporate alternative measures of biodiversity by taking into account distances (e.g., functional or phylogenetic) between pairs of species (De Bello et al., 2010).

We first calculated the total diversity of a sampling unit pooling local communities together (gamma‐diversity, *γ*_eqv_) and the average diversity of local communities (alpha‐diversity, *α*_eqv_) within a unit, and applied Jost's correction derived from equivalent numbers (Jost, 2007). Jost's correction was used to avoid ecologically meaningless results (see de Bello et al., 2010). We then calculated the beta‐diversity measure of interest,

$$\beta_{\text{prop}}=\frac{\gamma_{\mathrm{eqv}}-\alpha_{\mathrm{eqv}}}{\gamma_{\mathrm{eqv}}}$$

which represents the proportion of gamma‐diversity accounted for by the differentiation between local communities. Because gamma‐diversity is sensitive to variation in the number of local communities within a sampling unit, mere additive measure of beta‐diversity would be biased, but proportional measure of beta‐diversity (*β*_*prop*_) makes the sampling units with unequal number of local communities more comparable. In addition, using a proportional measure of beta‐diversity enabled us to directly compare the proportion of diversity explained by different facets of beta‐biodiversity (taxonomic, functional, and phylogenetic).

Taxonomic diversity measures were calculated using species abundance data collected from MNBBA point‐count locations. We calculated functional diversity by using numerical data on the diet composition (proportional use of 10 dietary categories), foraging niche traits (proportional use of eight categorical variables based on information on foraging stratum), activity time (ordinal from completely nocturnal to completely diurnal species) and body mass (in grams) from the dataset in Wilman et al., 2014 (for description of the data, see Belmaker & Jetz, 2013). Altogether, we used 20 functional traits, out of which 19 received values between 0 and 100 with 10‐unit intervals (diet composition, foraging stratum) or 20‐unit steps (activity time), and one was continuous (body size). Prior to analyses, all functional traits were scaled between 0 and 1 to give them all equal weight.

Functional distances were calculated with the daisy function in the R package cluster using Euclidean distances. Given that 18 out of 20 functional traits describe diet or foraging‐related variation among species, this variation will inevitably have much weight in the functional distance calculation. Functional distances were scaled between 0 and 1 and used as a distance matrix in the calculation of functional diversity metrics.

For the calculation of phylogenetic diversity, we downloaded a set of 1000 randomly chosen phylogenetic trees from the BirdTree database (Jetz et al., 2012), limited to the bird species found in our data. We used the “consense” function from the PHYLIP package (v.3.695) to create a single unrooted consensus tree using the 50% majority rule. This included all species that appeared in more than 50% of the trees. A phylogenetic distance matrix for all species in our data was calculated with the distTips function from the adephylo R package, using the sums of branch lengths. These distances were then scaled between 0 and 1 and used to calculate phylogenetic diversities.

### 2.3 Calculation of explanatory variables

Human influence was estimated using the human footprint index (HFI) developed by the Wildlife Conservation Society and the Center for International Earth Science Information Network (2005). The HFI uses nine datasets describing four proxies of human influence measured between 1995 and 2004: total human population size, human land‐use (i.e., land transformation to build‐up areas and agricultural land), accessibility (e.g., road density), and electrical power infrastructure (Sanderson et al., 2002). Each 1 km^2^ grid cell in the global dataset was given a value ranging from 0 (low level) to 10 (high level) for each dataset. The nine values for each grid cell were summed and for each global biome, the cell with the lowest value got a value of 0, and the cell with the largest value got a value of 100. The HFI data were clipped to the state of Minnesota, and the average HFI value for each unit was calculated. HFI values in our study area summarized human influence well, as seen in the correlation coefficients between HFI and total human population, road density, and cover of human land‐use classes (Appendix S1).

In addition to studying the impact of general human influence, we studied how changes in forest cover influenced biodiversity. In the state of Minnesota, areas experiencing forest change (habitat loss) and those with high human influence are clearly separate (Appendix S3a,b). We quantified forest change from the global forest change dataset developed by Hansen et al. (2013). The dataset uses Landsat data to identify stand‐replacing disturbances or the total removal of tree canopy cover within each pixel. Using these data, we were able to quantify gross forest loss and gain at an annual frequency between 2000 and 2014. For each unit, we calculated the percent cover of forest change between 2000 and 2014. However, there was no reliable way of systematically separating forest change caused by humans from change caused by natural events such as forest fires and storms. There were four units that had been significantly impacted by three large forest fires (Cavity Lake (2006), Ham Lake (2007), and Pagami Creek (2011) fires). Since the results from models run with and without the affected units did not change significantly, we decided to keep these units in the analyses. According to the data, there was no forest gain in the 287 units used in the statistical analyses. For the analyses, we created a gradient of forest loss by taking the opposite number of the original negative loss.

In addition to variables describing human‐caused changes in the environment, we also accounted for other factors known to impact biological diversity (Honkanen et al., 2010; Qian, 2010). Habitat diversity for each unit was quantified by calculating the mean alpha‐diversity of the 11 forested habitat classes within units (see LANDFIRE reclassification described above). Temperature and precipitation are both known to impact biodiversity but due to high variance inflation (Zuur et al., 2007) in the models where both variables were included, we decided to include only temperature. For each unit, we calculated the mean temperature measured between 1980 and 2010 from data created by the PRISM Climate Group (PRISM Climate Group, n.d.). Information about net primary production (NPP) was obtained from remotely sensed data collected by the MODIS instrument at a 1 km resolution. The algorithm that produces NPP values takes into account vegetation characteristics, meteorological measurements, and land cover class and estimates NPP as kg of carbon sequestered in the form of biomass per square meter per year (Zhao et al., 2005). For each unit, we averaged NPP values between 2004 and 2014.

We entered the number of forested MNBBA count locations per sampling unit as an independent variable to control its effects. Due to the sampling design of the Minnesota Breeding Bird Atlas the number of forested MNBBA count locations correlates with the % forest cover per sampling unit (*r* = 0.68). One may expect alpha, beta, and gamma‐diversities be sensitive to variation in forest cover (richer regional species pool and more variation among local communities), and thus including the number of forested MNBBA count locations into models also controls for the forest cover effects on diversity.

### 2.4 Statistical models

We constructed linear models with the lm function from the stats‐package (R Core Team, 2022) to analyze the influence of human footprint, forest loss, habitat diversity, temperature, NPP, and the number of forested MNBBA count locations on taxonomic, functional, and phylogenetic beta‐diversity (*β*_*prop*_). In addition, to aid in interpreting the results, we also analyzed the influence of the explanatory variables on alpha (*α*_*eqv*_) and gamma‐diversity (*γ*_*eqv*_) for the three facets of biodiversity. The results for gamma‐diversities can be found in Appendix S4.

We checked all models to ensure that the variance inflation factors (VIF) of the explanatory variables were <3 (Zuur et al., 2007) using the vif‐function from the car‐package (Fox & Weisberg, 2019). Correlations between explanatory variables can be found in Appendix S5 and VIF values for all models can be found in Appendix S6.

We visually inspected the residuals of all models to ensure that they were normally distributed, and tested residuals for spatial autocorrelation. All of the residuals from models explaining taxonomic diversity and models explaining alpha and gamma‐diversity for functional and phylogenetic diversity were normally distributed. The residuals for functional and phylogenetic beta‐diversity were right skewed and were thus modeled appropriately. In the case of functional beta‐diversity, we used a generalized linear model with Gamma family and an identity link using the glm function from the stats‐package (R Core Team, 2022). Spatial autocorrelation of residuals was tested with a permutation test for the Moran's I statistic using 1000 permutations with the moran.test function from the spdep‐package (Bivand, 2022). We detected spatial autocorrelation in the residuals of models explaining phylogenetic beta‐diversity, as well as all facets of alpha and gamma‐diversity. For cases where residuals were spatially autocorrelated, we utilized spatial error models, which is a type of autoregressive model. Spatial error models assume that spatia l autocorrelation is due to missing spatial covariates (Bivand & Piras, 2015). Spatial error models take into account spatial dependence in the error term of a spatial unit and its neighbors. These models were constructed using the errorsarlm function from the spdep‐package (Bivand, 2022). In the case of phylogenetic beta‐diversity, the residuals of the linear model were not normally distributed. Since the model we used was unable to utilize the generalized linear model framework, we log‐transformed the response variable and back‐transformed the results. The residuals from other facets of biodiversity analyzed using spatial models were normally distributed. We did not detect spatial autocorrelation in any of the residuals from the spatial error models. All analyses were performed with R version 3.5.1 (R Core Team, 2022). A list of R packages and their versions can be found in Appendix S7.

## 3 RESULTS

### 3.1 Alpha‐diversity

Taxonomic alpha‐diversity was not impacted by human footprint index (HFI), nor forest loss. Similarly, habitat diversity, net primary production, and mean temperature also were not related to taxonomic alpha‐diversity (Table 1).

**TABLE 1:** Summary of spatial error models explaining alpha‐diversities.

	Estimate	Std. error	z value	*p* value
Taxonomic alpha‐diversity				
Intercept	5.04	1.52	3.32	**.011**
Human footprint	−7.51*10^−3^	0.013	−0.598	.550
Forest loss (%)	−0.012	0.025	−0.489	.625
Net primary production (kg C/m^2^/Year)	3.01	2.36	1.27	.203
Habitat diversity	0.041	0.060	0.681	.496
Mean temperature (°C)	0.115	0.16	0.716	.474
Number forested points	−0.081	0.039	−2.09	**.037**
Functional alpha‐diversity				
Intercept	1.45	0.059	24.5	**<.001**
Human footprint	−6.12*10^−4^	4.92*10^−4^	−1.24	.214
Forest loss (%)	−2.46*10^−3^	9.74*10^−4^	−2.53	**.012**
Net primary production (kg C/m^2^/Year)	0.176	0.092	1.92	.055
Habitat diversity	0.003	0.002	1.37	.170
Mean temperature (°C)	0.018	0.006	2.97	**.003**
Number forested points	−0.005	0.002	−3.52	**<.001**
Phylogenetic alpha‐diversity				
Intercept	1.60	0.095	16.9	**<.001**
Human footprint	−1.53*10^−3^	8.06*10^−4^	−1.90	.058
Forest loss (%)	−3.35 *10^−3^	1.61*10^−3^	−2.07	**.038**
Net primary production (kg C/m^2^/Year)	0.151	0.145	1.04	.300
Habitat diversity	3.61*10^−3^	3.80*10^−3^	0.948	.343
Mean temperature (°C)	0.014	9.69*10^−3^	1.41	.158
Number forested points	−1.47*10^−3^	2.51*10^−3^	−0.588	.557

*Note*: Significant *p*‐values are in bold.

As predicted, functional alpha‐diversity was negatively associated with forest loss, but HFI was unrelated (Table 1). The association with mean temperature was positive. There also was a nearly significant positive association of net primary production with functional alpha‐diversity.

Phylogenetic alpha‐diversity was negatively associated with forest loss. Human footprint had nearly significant, albeit weak, negative relationship with phylogenetic alpha‐diversity, but net primary production, habitat diversity, or mean temperature had no relationship with phylogenetic alpha‐diversity (Table 1).

### 3.2 Beta‐diversity

**FIGURE 1:**
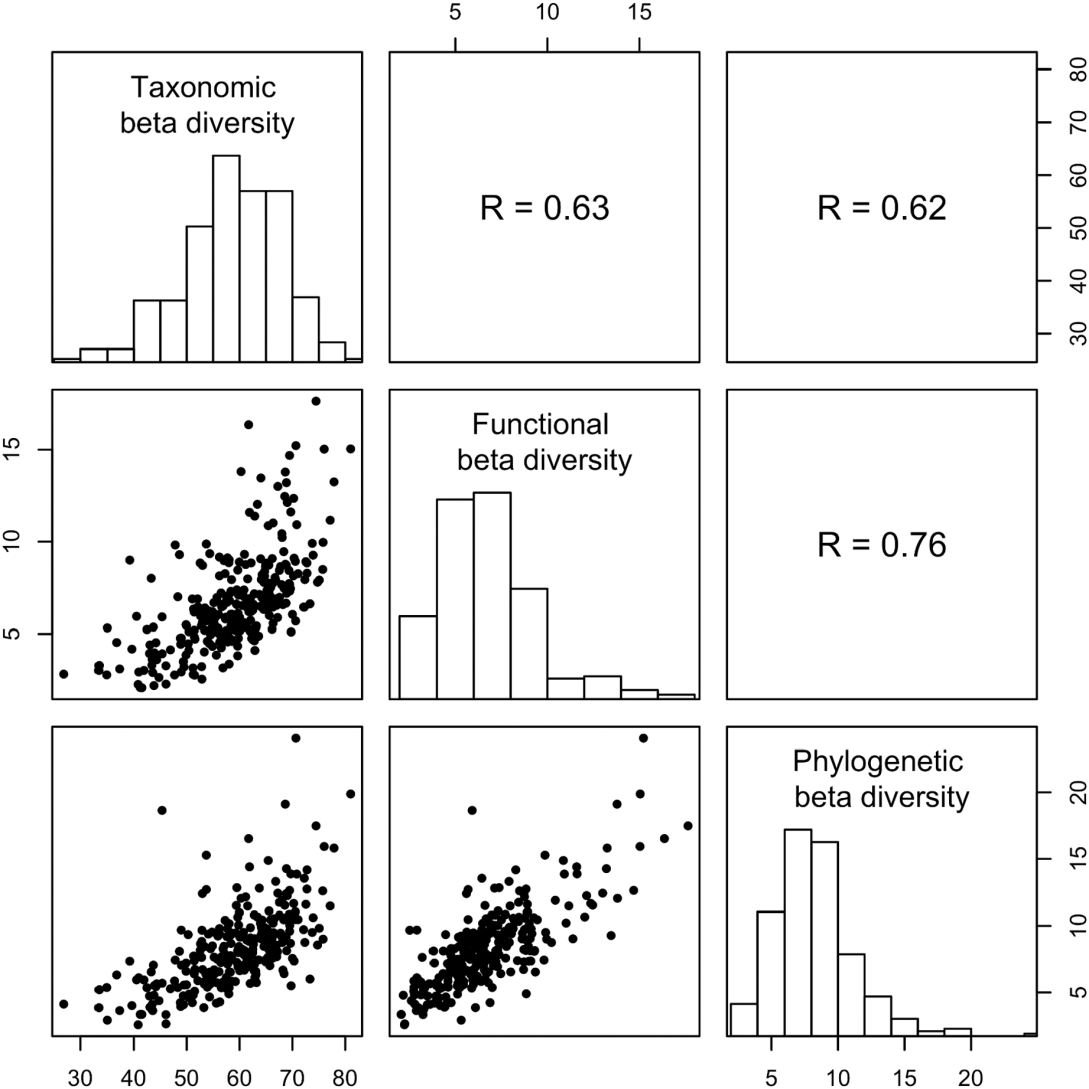
Distributions of taxonomic, functional and phylogenetic beta‐diversity, and Pearson correlations between the diversity measures.

Taxonomic, functional, and phylogenetic beta‐diversity measures were clearly correlated with each other, especially the correlation between functional and phylogenetic beta‐diversity (Figure 1). Spatial distribution of beta‐diversities is shown in Appendix S8.

Taxonomic beta‐diversity was unrelated to HFI and forest loss (Table 2). Instead, taxonomic beta‐diversity was positively associated with habitat diversity. Along the range of habitat diversity values (Appendix S5), taxonomic beta‐diversity increased by 6.9 units, which is a modest increase in biological terms (12.7%). This positive association was likely related to the positive influence of habitat diversity on taxonomic gamma‐diversity (Appendix S4A) and the lack of an association with taxonomic alpha‐diversity (Table 1).

**TABLE 2:** Summary of models explaining beta‐diversities.

	Estimate	Std. error	*t* value	*p* value
Taxonomic beta‐diversity				
Intercept	50.8	6.16	8.25	**<.001**
Human footprint	0.088	0.057	1.55	.122
Forest loss (%)	−0.040	0.118	−0.337	.737
Net primary production (kg C/m^2^/Year)	−13.2	9.18	−1.44	.151
Habitat diversity	0.661	0.257	2.57	**.011**
Mean temperature (°C)	−0.866	0.604	−1.44	.152
Number forested points	1.73	0.181	9.57	**<.001**
Functional beta‐diversity				
Intercept	10.5	2.02	5.23	**<.001**
Human footprint	0.047	0.0199	2.38	**.018**
Forest loss (%)	6.80*10^−3^	0.038	0.178	.859
Net primary production (kg C/m^2^/Year)	−9.72	3.02	−3.22	**.001**
Habitat diversity	0.016	0.086	0.188	.851
Mean temperature (°C)	−0.406	0.190	−2.13	**.034**
Number forested points	0.194	0.061	3.18	**.002**
Phylogenetic beta‐diversity				
Intercept	2.53	0.297	8.51	**<.001**
Human footprint	3.81*10^−3^	2.59*10^−3^	1.47	.141
Forest loss (%)	−2.49*10^−3^	5.26*10^−3^	−0.473	.636
Net primary production (kg C/m^2^/Year)	−1.10	0.453	−2.42	**.016**
Habitat diversity	3.44*10^−3^	0.012	0.284	.776
Mean temperature (°C)	−0.066	0.030	−2.20	**.028**
Number forested points	0.033	8.15*10^−3^	4.02	**<.001**

*Note*: For taxonomic and functional beta‐diversity we used generalized linear models and for phylogenetic beta‐diversity spatial error model. Significant *p*‐values are in bold.

Functional beta‐diversity had a significant positive association with HFI (Table 2). Along the gradient of HFI values in our data (Appendix S5), functional beta‐diversity increased by 3.1 units, which was a moderately strong biological impact (19.9%). HFI did not have a clear impact on functional alpha or gamma‐diversity but the negative estimate for functional alpha‐diversity was almost 30 times larger than the positive estimate for functional gamma‐diversity (Table 1, Appendix S4B). This might have resulted in the positive association of HFI and functional beta‐diversity (see ‘Section 4’). Forest loss did not appear to influence functional beta‐diversity. This was likely due to the negative association of forest loss with both functional alpha and gamma‐diversity.

Functional beta‐diversity had a strong negative association with net primary (NPP; Table 2). Along the range of NPP values (see Appendix S5), functional beta‐diversity decreased by 3.3 units (−21.2%), which was even stronger than the impact of HFI. Similarly, mean annual temperature was negatively associated with functional beta‐diversity (14.1% decrease along the gradient). Interestingly, both the impacts of NPP and temperature on functional beta‐diversity were not accompanied by straightforward associations on functional alpha and gamma‐diversity (Table 1, Appendix S4B). NPP appeared to have a positive but non‐significant association with alpha‐diversity but no association with gamma‐diversity, which is expected to result in a decrease in beta‐diversity with increasing NPP. On the other hand, mean temperature had a positive and similarly strong association with both functional alpha and gamma‐diversity, which is expected to result in no apparent association of beta‐diversity with mean temperature.

Similar to taxonomic beta‐diversity, phylogenetic beta‐diversity was not associated with HFI, nor forest loss (Table 2). This was despite the negative (albeit non‐significant) association of HFI with phylogenetic alpha‐diversity (Table 1) and no association with phylogenetic gamma‐diversity (Appendix S4C), which is expected to result in an increase in phylogenetic beta‐diversity with increasing HFI. However, the estimate of HFI's association with phylogenetic alpha‐diversity was only two times as large as the estimate for phylogenetic gamma‐diversity, compared with the large difference in estimates in the case of functional beta‐diversity. As was the case with functional diversity, forest loss had a negative association with both phylogenetic alpha and gamma‐diversity, which likely canceled out each other's effect on beta‐diversity, and we observed no effect of forest loss on phylogenetic beta‐diversity.

Phylogenetic beta‐diversity was negatively associated with NPP and mean temperature (Table 2). Along the range of NPP values (Appendix S5), phylogenetic beta‐diversity decreased by 6.7 units (−31%). Along the range of mean annual temperature values, phylogenetic beta‐diversity decreased by 6.5 units (−30.4%). NPP was not associated with either phylogenetic alpha (Table 1) or gamma‐diversity (Appendix S4C). Mean temperature had a positive relationship with phylogenetic alpha‐diversity, which was likely associated with the negative impact of temperature on phylogenetic beta‐diversity.

## 4 DISCUSSION

Elevated human pressure did not appear to be related to increased biotic homogenization in our study region. An increase in the human footprint index, which measured the impact of increased population density, land transformation, accessibility, and electrical power infrastructure, did not cause biotic homogenization in taxonomic, functional, or phylogenetic diversity. Instead, increased human footprint appeared to be related to an increase in functional heterogeneity, and therefore, increasing functional diversity among sites. As such, our results appear to be at odds with previous studies that have shown homogenizing impacts of increased human pressure. For example, urbanization has been shown to homogenize environments and, consequently, biological communities (McKinney, 2006; Sol et al., 2017), and agricultural expansion and intensification have been shown to homogenize bacterial (Rodrigues et al., 2013), beetle (Gordon et al., 2009), and grassland bird communities (Liang et al., 2019). Others have shown homogenization to occur with the human footprint in biological communities, especially in the tropics (Ibarra & Martin, 2015; Kitching et al., 2013; Lôbo et al., 2011). However, it is important to note that some studies have shown no biotic homogenization in response to increased human pressure (Lee‐Cruz et al., 2013) or, like this study, even an increase in beta‐diversity (Catterall et al., 2010; Fugère et al., 2016; Roa‐Fuentes et al., 2019).

The result that increased human footprint appeared to be associated with increased functional diversity between sites is contrary to the results of many previous studies (e.g., McKinney, 2006). Human footprint clearly summarized human pressures well since it was strongly correlated with total population, density of roads, and human land‐use cover (Appendix S1). A potential reason for beta‐diversity increases in response to increased human impact is likely related to the calculation of beta‐diversity. Beta‐diversity, in both its additive and multiplicative forms, is derived from total regional diversity (gamma‐diversity) and mean local diversity (mean alpha‐diversity). We calculated beta‐diversity as gamma‐diversity minus mean alpha‐diversity. Thus, increase in beta‐diversity could be associated with an increase in gamma‐diversity or a decrease in alpha‐diversity, or both. Human footprint did not have a statistically significant impact on functional gamma or alpha‐diversity but the negative estimate for local diversity was more than an order of magnitude stronger than that for regional diversity, which then resulted in the positive impact on beta‐diversity. The negative estimate for the impact of human footprint on functional alpha‐diversity suggests that increased human footprint has led to a loss of functionally distinct species from local communities. This is in line with Bracey et al. (2022) who found that functional alpha‐diversity declined significantly with increasing human land use. If this loss occurred randomly, impacting some areas but not others, this could lead to MNBBA count locations being more different from each other. This loss of species from local communities with increasing human footprint suggests that functional beta‐diversity may have primarily been affected by the nestedness component of beta‐diversity, although impacts on turnover resulting from species replacement along the human footprint gradient cannot be ruled out (Baselga, 2010).

Devictor et al. (2008) demonstrated that the functional homogenization of bird communities is strongly positively correlated to landscape disturbance and fragmentation. Fragmentation is not likely a key factor in our study area because northern Minnesota is still a mostly forested region. Only about 10% of the sampling units have percentage forest cover lower than 20% (typically considered a critical threshold for fragmentation effects to appear; Mönkkönen & Reunanen, 1999).

The positive response of beta‐diversity to increasing human pressure is supported by the conceptual trajectory outlined in Socolar et al. (2016). In this model, beta‐diversity first increases with increasing human impact in response to subtractive heterogenization caused by some native species becoming rarer and invasive species beginning to establish themselves (additive heterogenization). With further increase in human impact beta‐diversity begins to decrease as rarer species begin to disappear and invasive and/or generalist species start to dominate. Since we only used relatively forested units in our analysis the level of human pressure in these units was clearly lower than that of units left out of analyses (Appendix S9). Therefore, it is possible that human pressure in our study was so low that it increased diversity between MNBBA sites. In our data, there was only one truly invasive species, the European starling. This species was more common in units with higher HFI (near towns/farms).

Forest loss and human footprint were clearly spatially segregated in the state of Minnesota (Appendix S1), and thus captured different facets of human pressure in the state. However, like human footprint, forest loss did not appear to cause biotic homogenization in any of the response variables. Forest management has also been shown to cause biotic homogenization of forest communities, although the impacts have often been subtle (Ibarra & Martin, 2015; Kitching et al., 2013; Mori et al., 2015). Häkkilä et al. (2018) found signs of homogenization in protected areas surrounded by intensively managed forest, although the quality of habitats within the protected areas played a more substantial role than the surrounding landscape in determining species composition. A loss in forest cover did, however, appear to decrease functional and phylogenetic diversity at the local (alpha‐diversity) and regional (gamma‐diversity) scales, although the impact on regional functional diversity was not statistically significant. Since forest loss had a negative impact at both local and regional scales, the method of calculating beta‐diversity would have canceled out the impact of forest loss on beta‐diversity, meaning that no homogenizing effects of forest loss were observed. This suggests that even though units lost local and regional diversity in response to forest loss, MNBBA sites did not become more alike.

It is possible that the forests in this region, which have experienced heavy logging of natural forests over the past 100–150 years (Schulte et al., 2007), have already been homogenized in terms of species composition and forest structure to such an extent that current forest loss and management activities occurring have limited impact on the composition of bird communities. Indeed, forests in the area still bear a clear signature of previous land‐use change (Schulte et al., 2007). Prior to continuous European settlement around 1850, there was approximately 12.8 million ha of forest land in Minnesota with about half lost from land clearing by logging and burning for agriculture and settlement by the early 1900s.

Evaluations of the effects of 150 years of forest loss in Minnesota on bird species distribution (Frelich & Niemi, 2021; Pfannmuller et al., 2017, 2023; Schulte et al., 2005) documented forest range contractions for many species of birds. These documented range contractions most assuredly were due to loss of forests and their conversion to agriculture, urban, and exurban areas. Overall, the forest bird communities within the area studied are largely intact, albeit distributions and abundances for many forest species are reduced. However, the forested areas included in this study did not include most of those areas that have been converted such as in southern Minnesota. Had we focused on all bird species or specifically on birds in open habitats—and consequently considered the regions in Minnesota where the overall human impact is strong—the results would likely have been different.

It was impossible to separate forest loss caused by humans (e.g., clear cutting) from change caused by natural events such as forest fires and storms. Even though logging is still an important disturbance type in northern Minnesota many other, climate change‐related, disturbance factors such as diseases, insect infestations, wind, fire, and drought have increasingly shaped Minnesota's forests (Wilson et al., 2019). Future studies would benefit from more direct estimate of logging intensity than was available for our study and could more specifically aim at discerning between the effects of human logging disturbances and other disturbances.

In addition to studying the impacts of variables that captured human pressures on beta‐diversities, we also considered three environmental variables known to influence patterns of biodiversity: habitat diversity, net primary production, and average temperature (Qian, 2010). Habitat diversity had a clear positive association with taxonomic beta‐diversity, meaning that units with a higher diversity of forest habitat types tended to have bird communities that were different from each other. This influence, as one may expect, was due to an increase in taxonomic gamma‐diversity with increasing habitat diversity (Appendix S4A). Net primary productivity and average temperature did not influence taxonomic beta‐diversity, but both had a negative association with functional and phylogenetic beta‐diversity. In the case of functional beta‐diversity, both variables appeared to negatively influence beta‐diversity by increasing the diversity within MNBBA sites, which would lead to a decrease in beta‐diversity. In the case of phylogenetic beta‐diversity these variables did not appear to be associated with an increase in diversity within MNBBA sites. The relatively strong correlation between functional and phylogenetic beta‐diversity (Figure 1) suggests that the same cause was behind the negative influence of productivity and temperature on phylogenetic beta‐diversity.

## 5 CONCLUSIONS

Ultimately, conservation actions aim at maintaining global and regional diversity. High regional diversity can be achieved either by consistently high local diversities or by high beta‐diversity. From a conservation viewpoint, maximization of beta‐diversity, that is, increasing differences in diversity between sites, is not necessarily desirable. Ensuring that local communities host different species could actually minimize species persistence over the long term because each species would be represented by a single or only a few local populations, resulting in higher rates of regional extinctions. In addition, it would be difficult to argue for increasing beta‐diversity at the expense of decreasing local diversity (Socolar et al., 2016) because targeting high average local diversity is a cost‐efficient conservation strategy particularly when nestedness in community composition is marked. Our results indeed suggest that increasing functional beta‐diversity with increasing human footprint can be due to a loss of functional diversity from local communities. This highlights the importance of interpreting with care in a conservation setting the results concerning beta‐diversity.

Our study supports earlier findings (e.g., Devictor et al., 2010; Häkkilä et al., 2017) that it is important to consider multiple facets of biodiversity. The positive association between increasing human footprint and biodiversity in our study region would have been missed had we only focused on taxonomic diversity, as has often previously been the case. Similarly, impacts on between‐site and regional diversity would have been missed if we had only studied local diversity. Our results suggest that increased human footprint and forest loss within the forest area studied and at the current time have not caused biotic homogenization in Minnesotan forests. Yet, with the growing human population and predicted changes in these forests due to climate change (Frelich & Reich, 2010), and given the observed staggering decline of forest bird populations in North America (Rosenberg et al., 2019), this result is likely temporary.

## AUTHOR CONTRIBUTIONS

**Eric Le Tortorec:** Conceptualization (equal); data curation (equal); formal analysis (lead); methodology (lead); writing – original draft (lead); writing – review and editing (equal). **Matti Häkkilä:** Conceptualization (equal); data curation (supporting); methodology (supporting); writing – original draft (supporting); writing – review and editing (equal). **Edmund Zlonis:** Data curation (equal); formal analysis (supporting); methodology (supporting); writing – original draft (supporting); writing – review and editing (equal). **Gerald Niemi:** Conceptualization (equal); data curation (equal); writing – original draft (supporting); writing – review and editing (equal). **Mikko Mönkkönen:** Conceptualization (equal); resources (lead); writing – original draft (supporting); writing – review and editing (equal).

## CONFLICT OF INTEREST STATEMENT

No competing interests.

## Supporting information


Appendix S1:

## Data Availability

R‐code and data for replicating statistical analyses used in the study are deposited in University of Jyväskylä JYX Publication Archive (DOI: https://doi.org/10.17011/jyx/dataset/86262).
