# Supplementary material for: Increasing human environmental footprint does not lead to biotic homogenization of forest bird communities in northern USA
Source: Ecol Evol. 2023 Apr 19;13(4):e10015. doi: 10.1002/ece3.10015 (PMC10116078; doi:10.1002/ece3.10015)

**Appendices**

**Appendix** **1. Correlations between human footprint and variables describing human influence.** Total human population, road density and human land were available as separate datasets and are plotted here against human footprint.


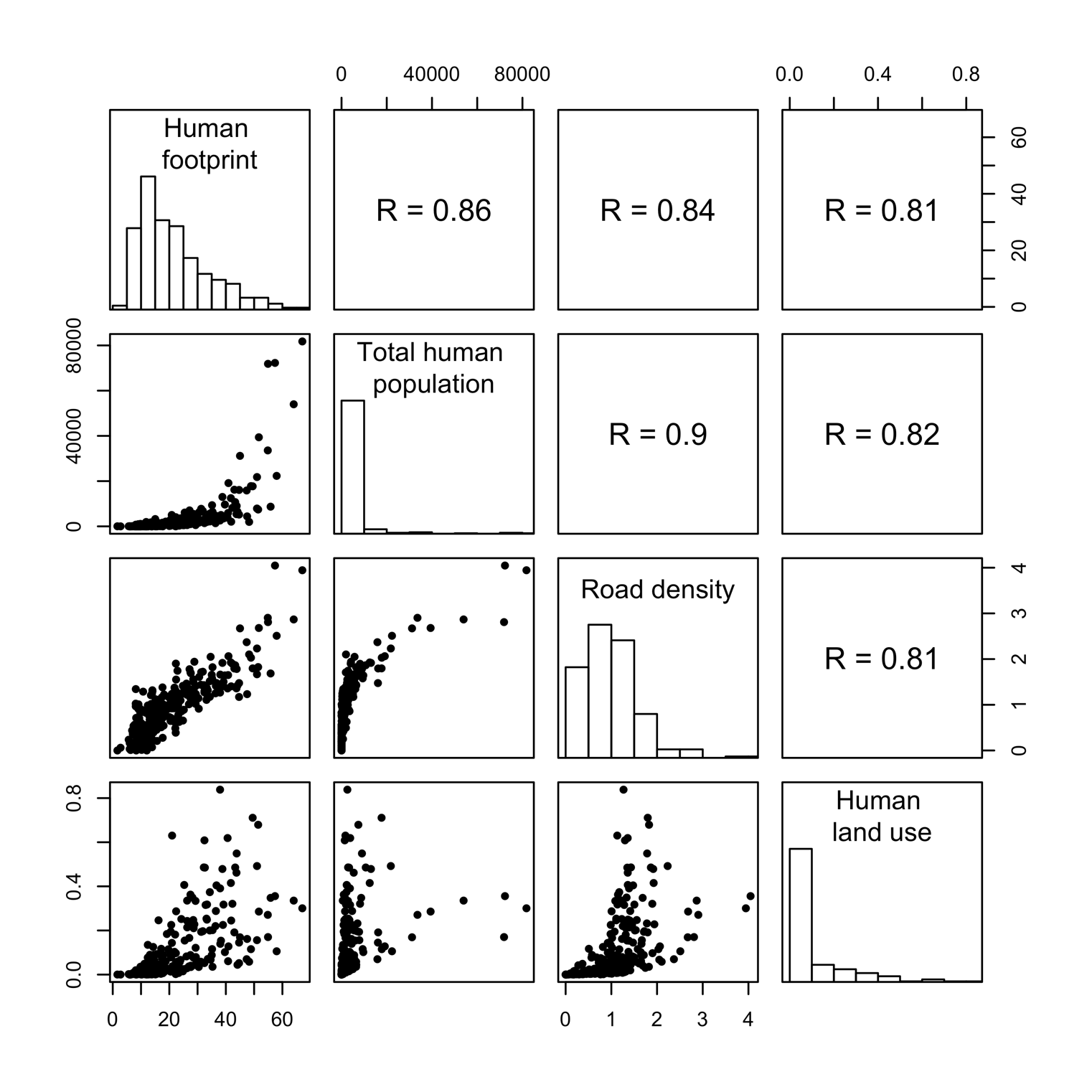


**Appendix 2. LANDFIRE classes classified as forests**

Boreal Coniferous

Boreal Deciduous

Lowland Coniferous Forest

Lowland Deciduous Forest

Northern Hardwoods

Oak Forest

Oak Savannah

Parkland Deciduous Forest

Pine Forest

Pine-Oak Barrens

Rural and Urban Developed Forest

## Appendix 3. Spatial distribution of explanatory variables in Minnesota.

### a) Human footprint


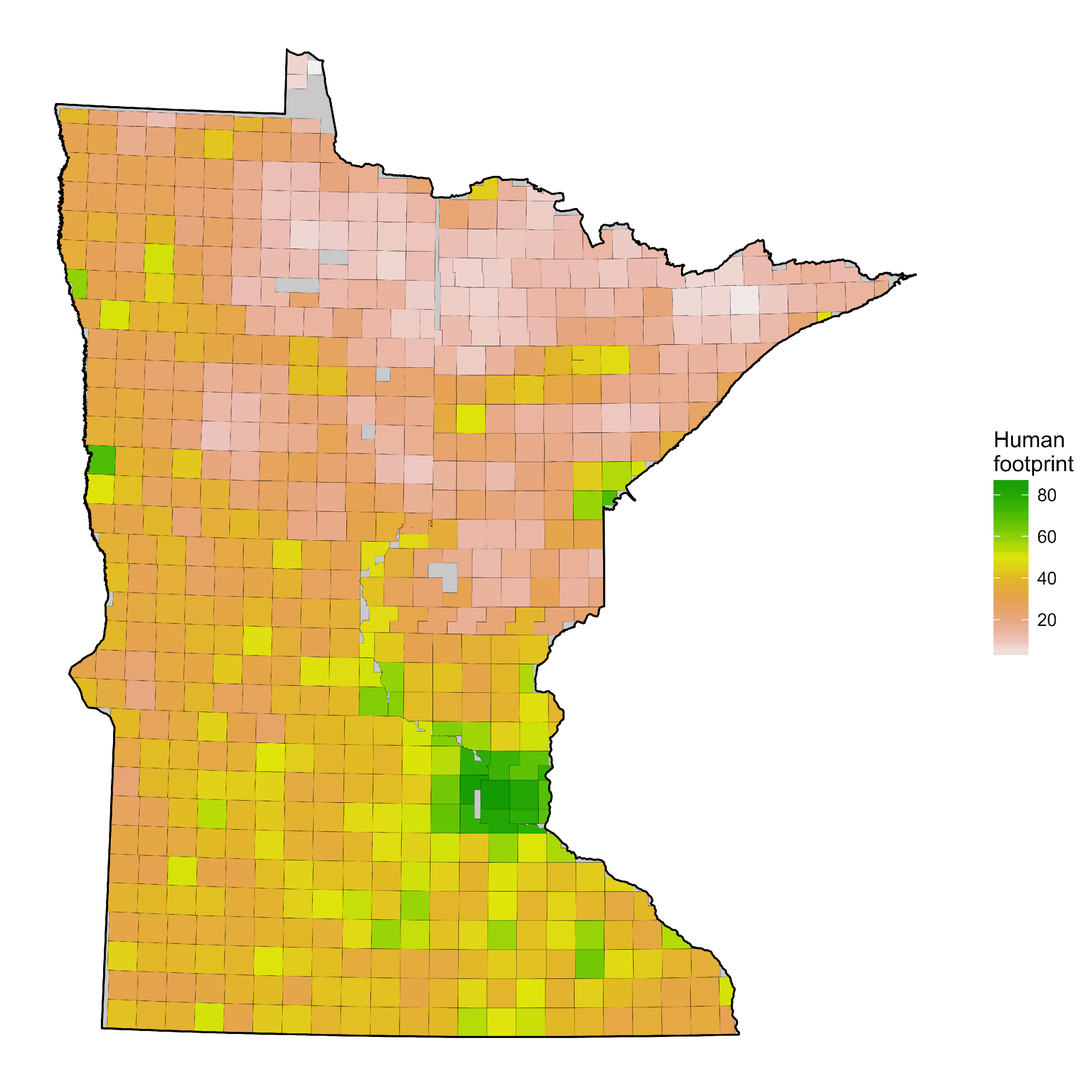


### b) Forest loss.


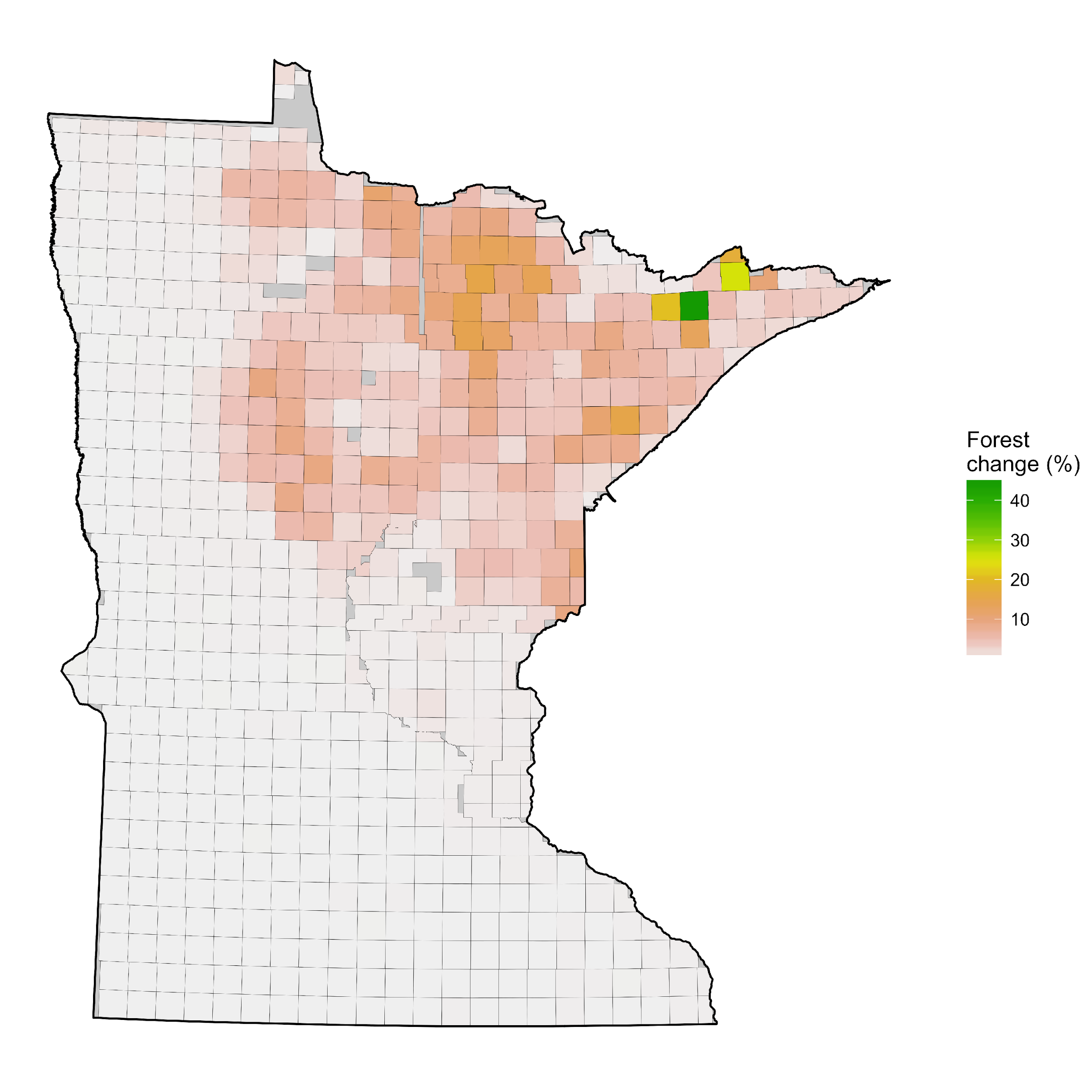


### c) Habitat diversity


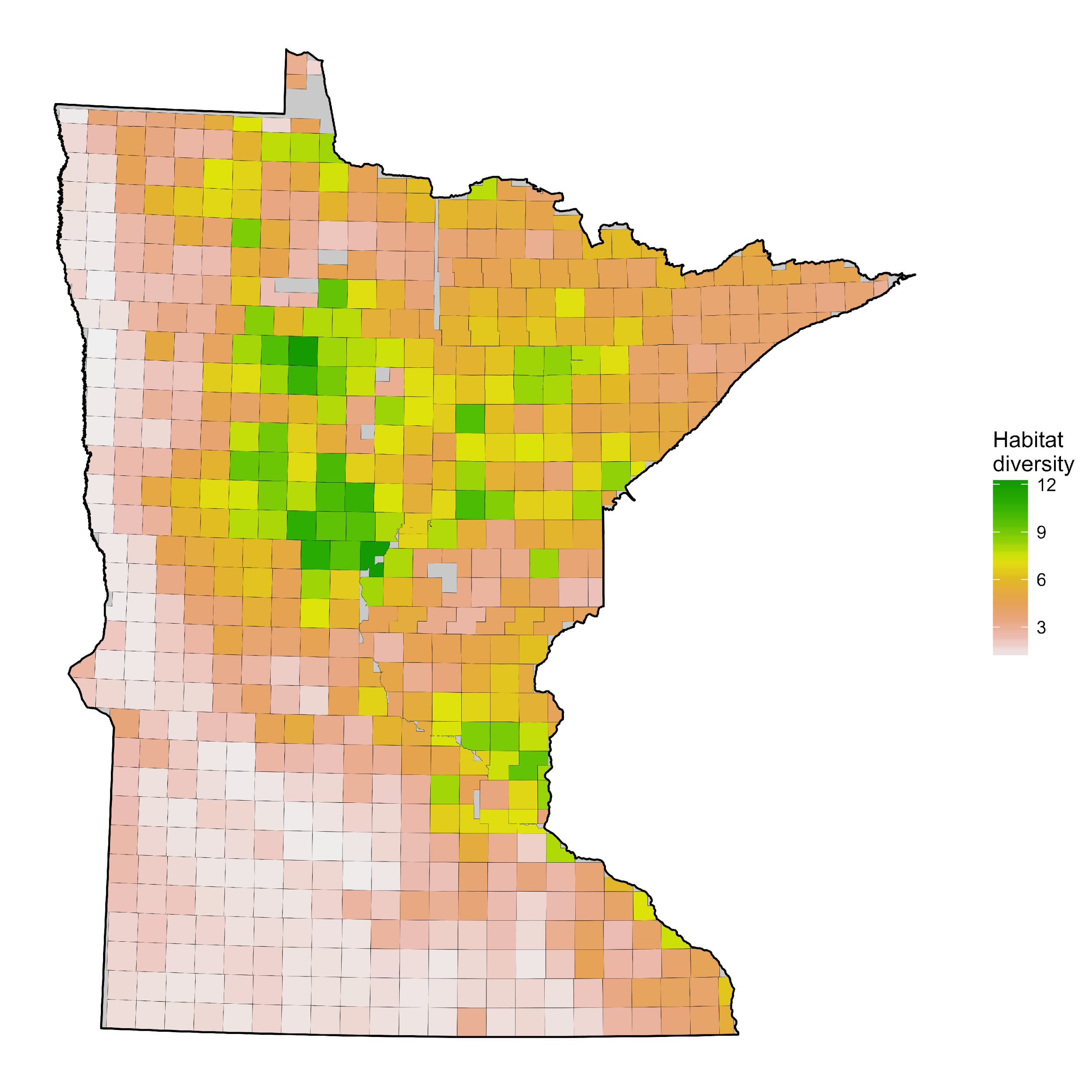


### d) Net primary production


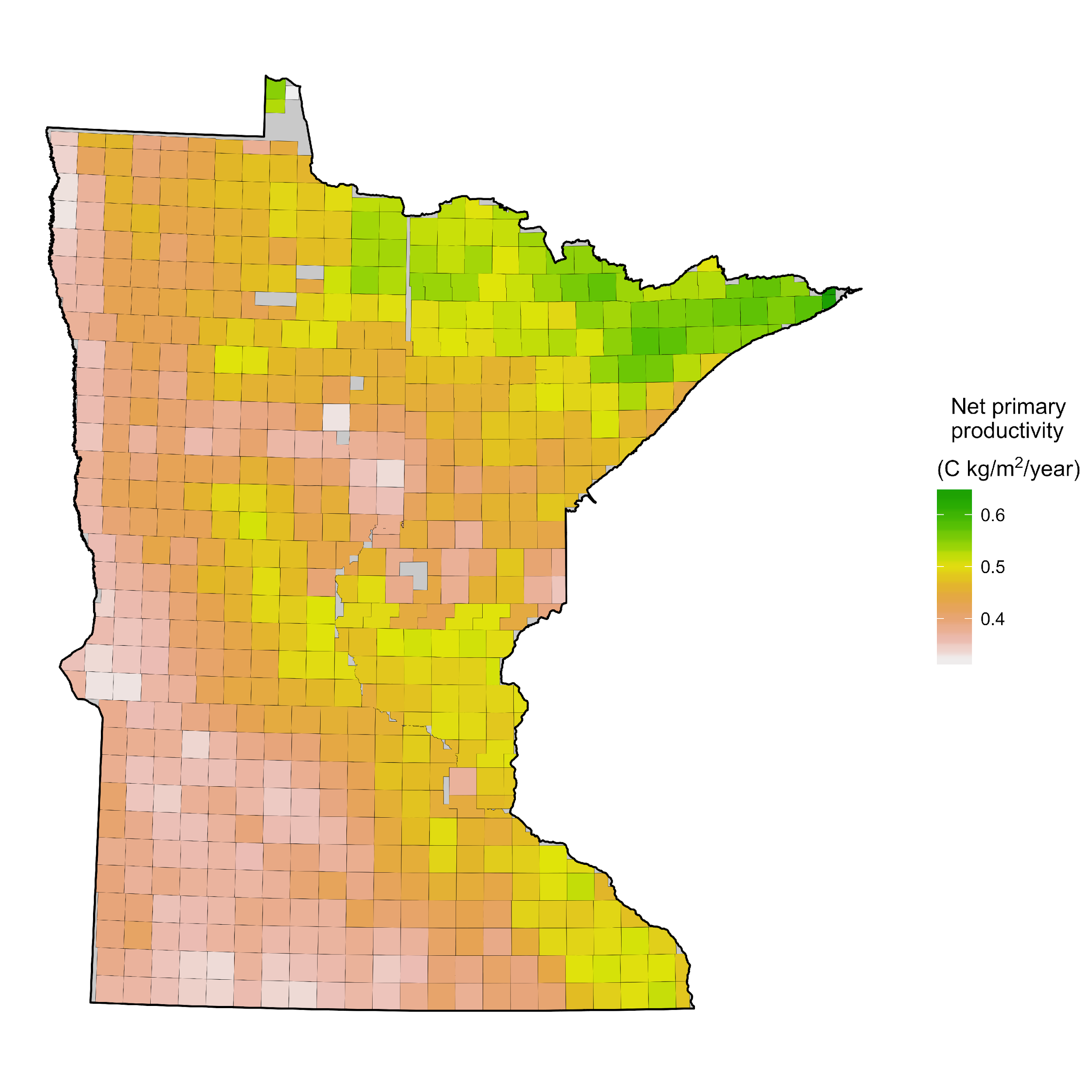


### e) Mean temperature


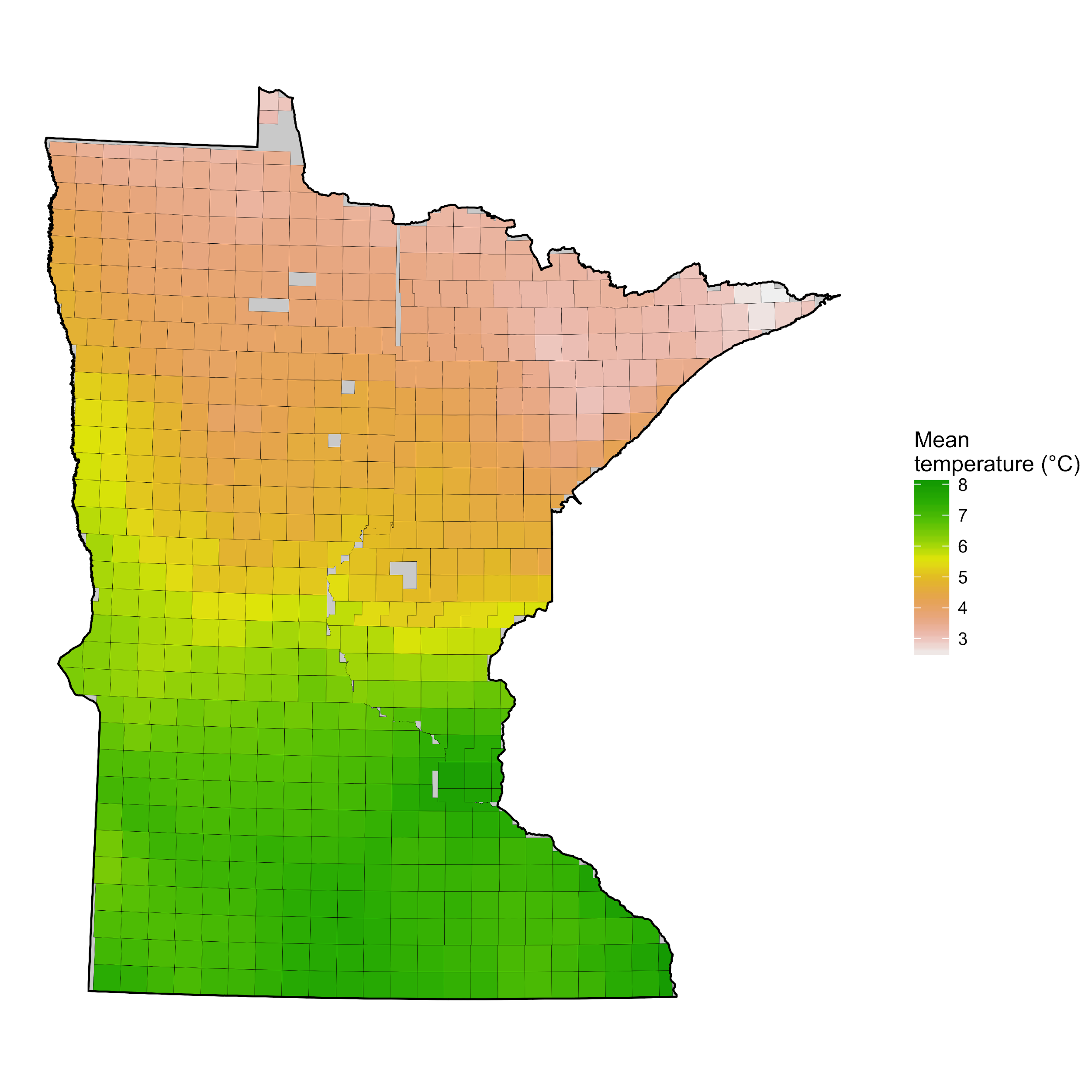


**Appendix** **4. Results of gamma diversities**

**Table 4A:** Summary of spatial error model explaining taxonomic gamma diversity. Statistically significant effects are in bold.

  Estimate Std. Error z value p value

**Intercept** 8.98 3.28 2.74 **0.006**

**Human footprint** 0.026 0.0274 0.935 0.350

**Forest loss (%)** 4.85e-3 0.054 0.089 0.929

**Net primary production (kg C/m^2^/Year)** 2.97 5.09 0.584 0.559

**Habitat diversity** 0.297 0.131 2.27 **0.023**

**Mean temperature (℃)** -0.076 0.342 -0.221 0.825

**Number forested points** 0.493 0.085 5.83 **5.45e-09**

**Table 4B:** Summary of spatial error model explaining functional gamma diversity

  Estimate Std. Error z value p value

**Intercept** 1.60 0.057 28.2 **0**

**Human footprint** 2.17e-05 4.78e-4 0.046 0.964

**Forest loss (%)** -1.96e-3 9.52e-4 -2.05 **0.040**

**Net primary production (kg C/m^2^/Year)** 0.041 0.088 0.467 0.641

**Habitat diversity** 3.55e-3 2.27e-3 1.56 0.118

**Mean temperature (℃)** 0.015 5.87e-3 2.56 **0.011**

**Number forested points** -2.34e-3 1.48e-3 -1.58 0.115

**Table 4C:** Summary of spatial error model explaining phylogenetic gamma diversity

  Estimate Std. Error z value p value

**Intercept** 1.85 0.096 19.2 **0**

**Human footprint** -7.85e-4 8.42e-4 -0.932 0.351

**Forest loss (%)** -3.86e-3 1.71e-3 -2.26 **0.024**

**Net primary production (kg C/m^2^/Year)** -0.039 0.147 -0.267 0.790

**Habitat diversity** 3.62e-3 3.93e-3 0.921 0.357

**Mean temperature (℃)** 2.24e-3 9.72e-3 0.230 0.818

**Number forested points** 2.33e-3 2.65e-3 0.880 0.379

**Appendix 5. Correlations between explanatory variables, and their distributions.** The number of point count locations denotes the number of forested MNBBA count locations per sampling unit.


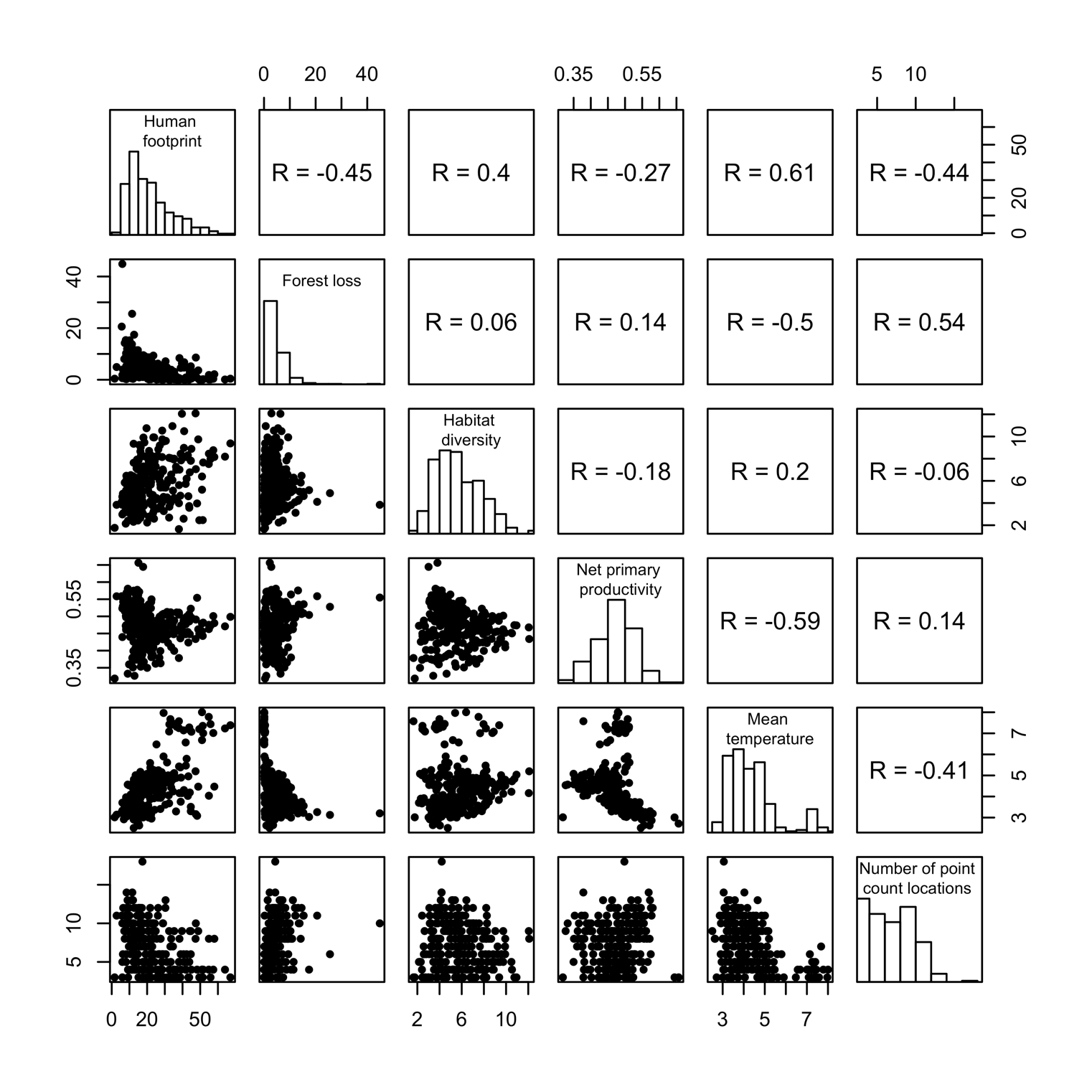


**Appendix 6.** *Variance inflation factors of explanatory variables in models explaining taxonomic, functional, and phylogenetic beta-diversity.*

|  | Human footprint | Forest change (%) | Net primary production (kg C/m^2^/Year) | Habitat diversity | Mean temperature (℃) | Number forested points |
| --- | --- | --- | --- | --- | --- | --- |
| **Taxonomic** | 2.37 | 1.34 | 1.21 | 1.29 | 2.29 | 1.36 |
| **Functional** | 2.64 | 1.35 | 1.21 | 1.28 | 2.54 | 1.36 |
| **Phylogenetic** | 2.37 | 1.34 | 1.21 | 1.29 | 2.29 | 1.36 |

**Appendix 7. List of used R packages and their versions**

base 3.5.1

datasets 3.5.1

dplyr 0.7.6

forcats 0.3.0

ggplot2 3.0.0

graphics 3.5.1

grDevices 3.5.1

here 0.1

methods 3.5.1

pander 0.6.2

printr 0.1

purrr 0.2.5

readr 1.1.1

sp 1.3.1

spdep 0.8.1

stats 3.5.1

stringr 1.3.1

tibble 1.4.2

tidyr 0.8.1

tidyverse 1.2.1

utils 3.5.1

**Appendix 8. Spatial distribution of response variables and species richness.** Grey color indicates areas (sampling units) excluded from this study due to too low number of forested MNBBA points**.**

a) Taxonomic beta diversity


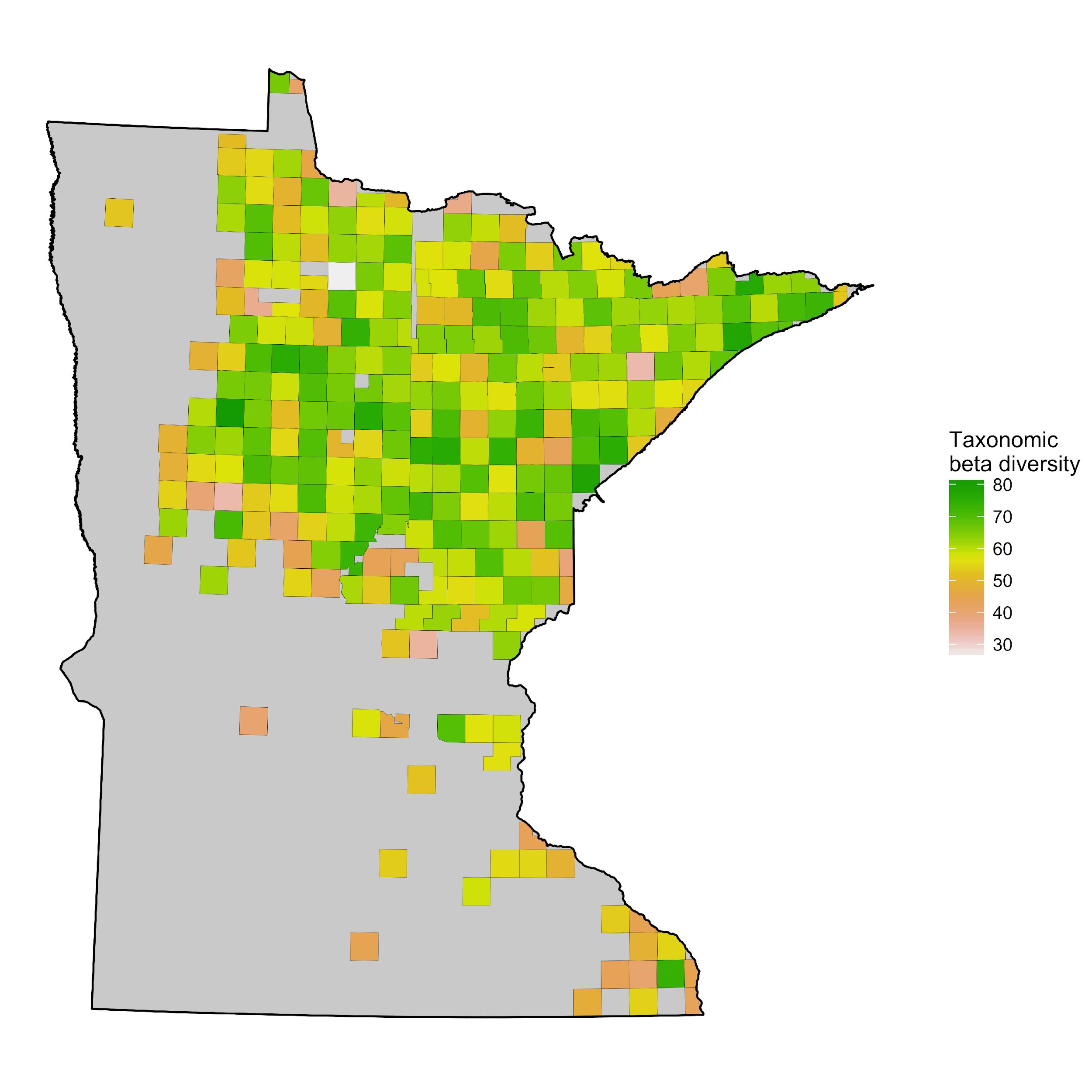


b) Functional beta diversity


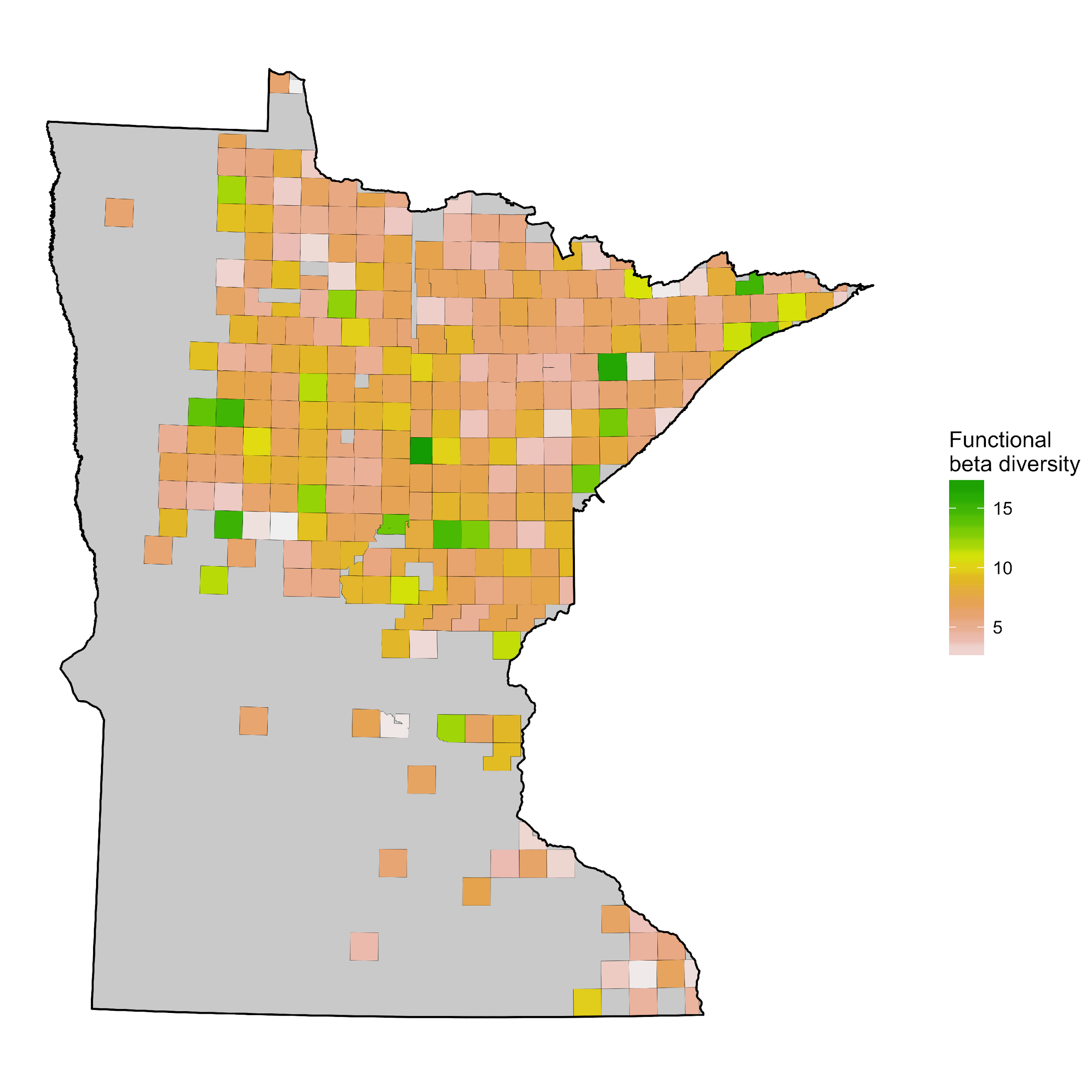


c) Phylogenetic beta diversity


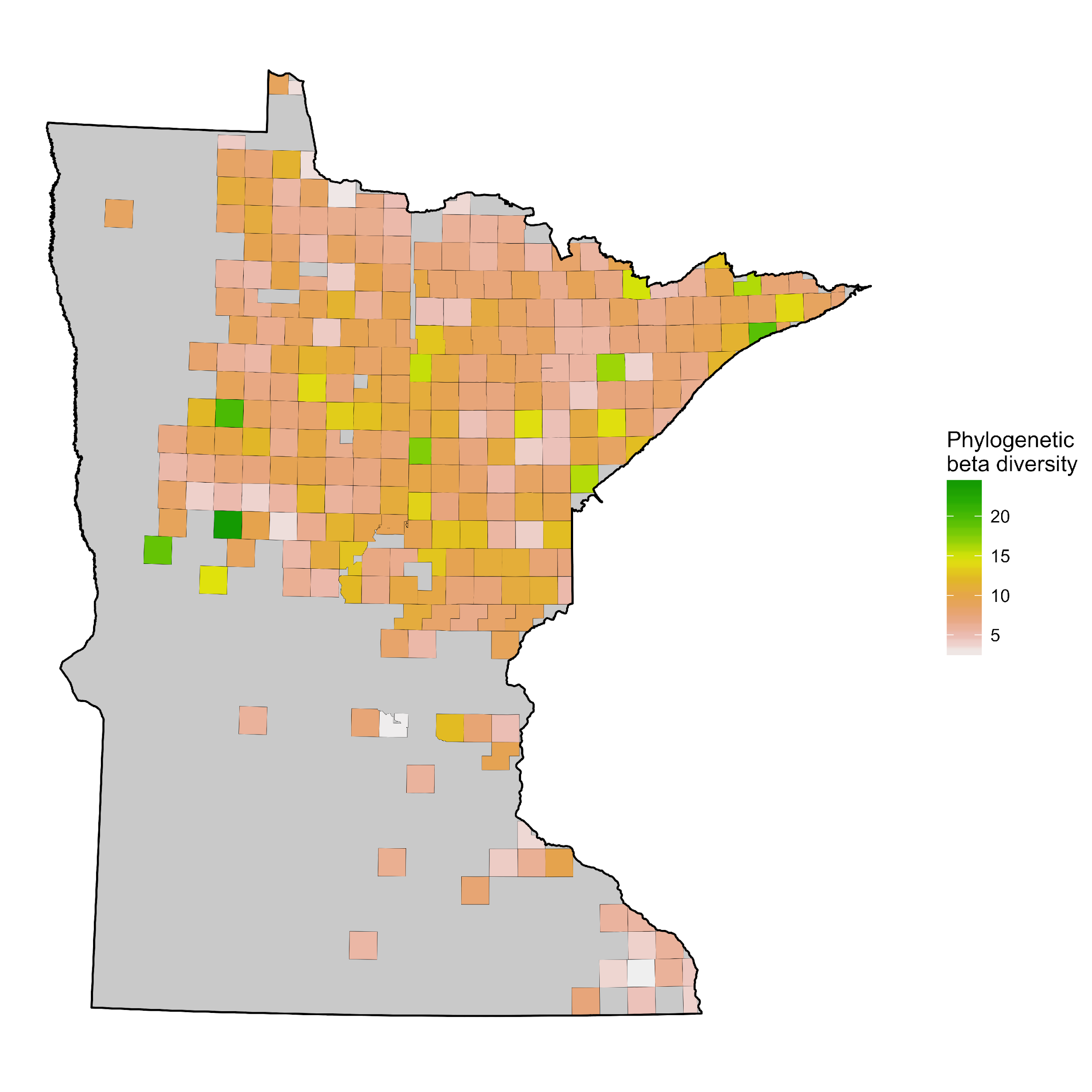


**Appendix 9.** Comparison of human footprint index values in units used in analyses (coloured areas in Appendix 8) and units not used in analyses (grey areas in Appendix 8). See Appendix 3 for spatial distribution of human footprint index values.


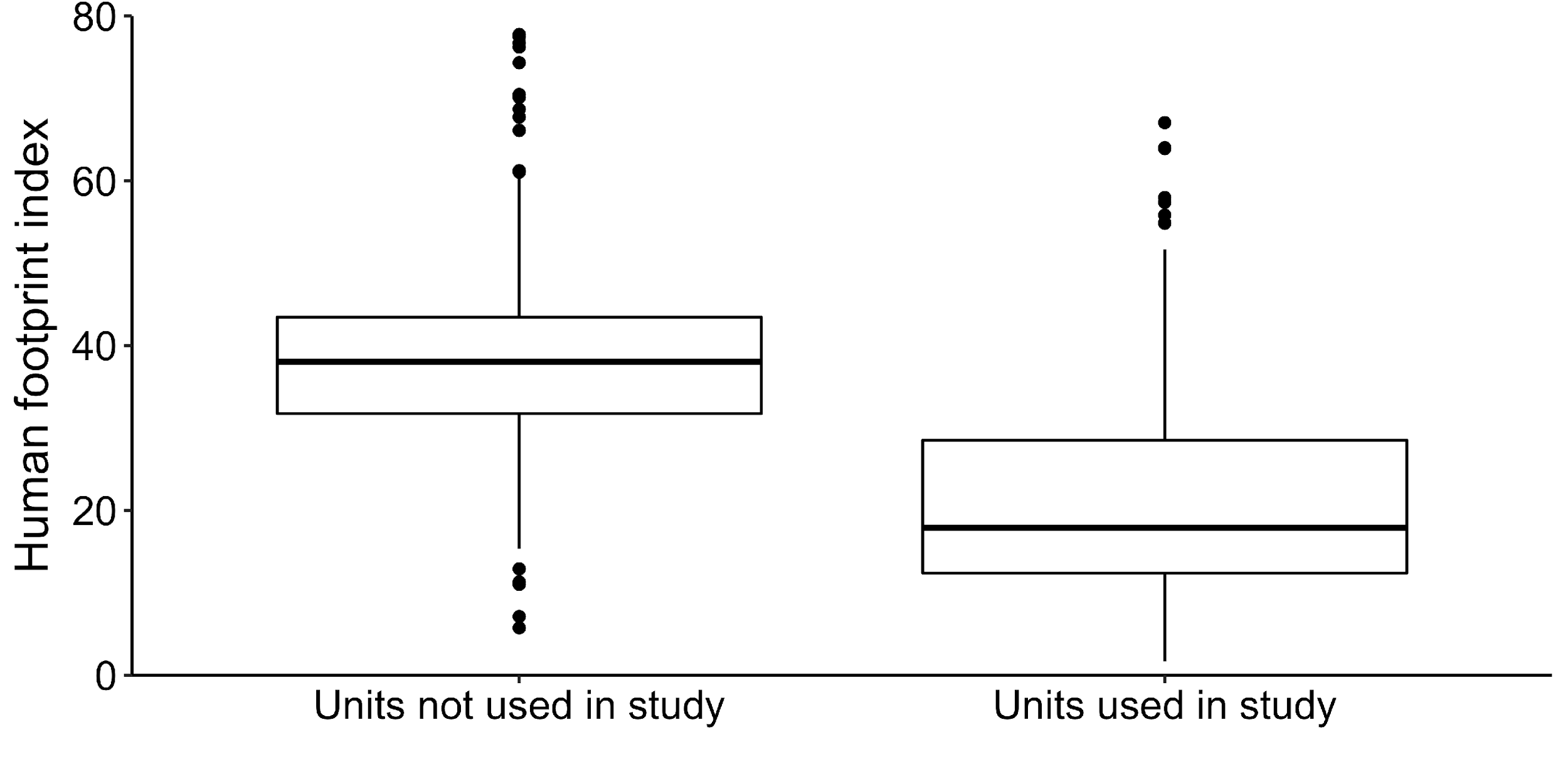

Supplement: Supplementary file 1 — Appendix S1: [file ECE3-13-e10015-s001.docx]
